# Effects of the Parent Alloy Microstructure on the Thermal Stability of Nanoporous Au

**DOI:** 10.3390/ma15196621

**Published:** 2022-09-23

**Authors:** Andrea Pinna, Giorgio Pia, Roberta Licheri, Luca Pilia

**Affiliations:** Department of Mechanical, Chemical and Materials Engineering, University of Cagliari, Piazza d’Armi, 09123 Cagliari, Italy

**Keywords:** nanoporous metals, coarsening, microstructure, dealloying, Ag-Au alloy, thermal stability, grain size, nanostructure

## Abstract

Nanoporous (NP) metals represent a unique class of materials with promising properties for a wide set of applications in advanced technology, from catalysis and sensing to lightweight structural materials. However, they typically suffer from low thermal stability, which results in a coarsening behavior not yet fully understood. In this work, we focused precisely on the coarsening process undergone by NP Au, starting from the analysis of data available in the literature and addressing specific issues with suitably designed experiments. We observe that annealing more easily induces densification in systems with short characteristic lengths. The NP Au structures obtained by dealloying of mechanically alloyed AuAg precursors exhibit lower thermal stability than several NP Au samples discussed in the literature. Similarly, NP Au samples prepared by annealing the precursor alloy before dealloying display enhanced resistance to coarsening. We suggest that the microstructure of the precursor alloy, and, in particular, the grain size of the metal phases, can significantly affect the thermal stability of the NP metal. Specifically, the smaller the grain size of the parent alloy, the lower the thermal stability.

## 1. Introduction

During the last decades, NP materials have attracted interest from many areas related to advanced technology. This is mostly due to the unique physical and chemical properties stemming from their large surface area, which gives rise to unique structural behavior and surface chemistry [1,2]. Within the wide class of NP materials, NP metals deserve a special mention [3,4,5]. They are commonly defined as metallic materials with pore size smaller than 200 nm and porosity higher than 30% [5]. Commonly produced by the dealloying of precursor alloys, they show a characteristic bicontinuous structure of interconnected metal ligaments and pores [3,4,5]. On the one hand, the monolithic form allows easy handling, also avoiding agglomeration and removing any need of suspension media [3,4,5]. On the other hand, NP metals can exhibit physical and chemical properties typical of metal nanoparticles. Therefore, it is not surprising that NP metal foams have been taken into consideration for structural applications [6,7], catalysis, electrocatalysis and supercapacitors [4,8,9], actuation [9], surface-enhanced Raman spectroscopy (SERS) and metal-enhanced fluorescence (MEF) [10,11,12,13], and electrochemical and optical sensing [14].

The sensitivity of NP metals to coarsening adds interest in the class of materials. Ligament and pore sizes can be, indeed, tuned as a function of processing parameters related to dealloying, thermal treatments, and the chemical environment [15,16,17,18]. The fine-tuning of the characteristic lengths from a few nm to tens of µm paves the way to the design of materials with properties optimized for specific applications. At the same time, it is worth noting that it is highly desirable to have NP metals with nanometric characteristic lengths able to work at high temperatures or during long electrochemical cycles of operation. Any significant increase of temperature can rapidly induce the increase of the characteristic lengths, with a consequent dramatic worsening of performances, for instance, in catalytic activity, SERS and MEF efficiency, and mechanical resistance. For this reason, it is of paramount importance to better understand the mechanisms underlying coarsening.

In this work, we focus on NP Au, the typical system used to investigate NP metal properties. NP Au is commonly produced by the dealloying of Au-Ag parent alloys. One of the open questions is that the properties of NP metals are influenced by many parameters, which often defy the description of experimental procedures. It is therefore difficult to identify the reasons behind different behaviors and properties between NP metal samples obtained under apparently similar experimental conditions. To a first approximation, coarsening is mediated by a surface diffusion process that makes NP Au evolve in a self-similar way without densification [19]. However, several reports show that thermal treatments can also lead to non-self-similar structures, with partial densification or even with a complete collapse of the porous structure [20,21,22]. Moreover, the evolution of ligament and pore sizes can greatly vary from one study to another [22,23]. In this work, we attempt to understand the factors that bring about such different behaviors. To this aim, we carry out an image analysis (IA) of figures showing NP Au structures reported in the literature and complete the framework with data coming from original experimental work. Particular attention is paid to the effects of different shapes and microstructures of the precursor alloys used for the NP Au fabrication.

Typically, NP Au is fabricated using commercially available monolithic Au-Ag alloys or starting from powders that are subjected to melting, casting, and subsequent annealing for homogenization and residual stresses relief. These standard procedures commonly result in grain sizes that range from a few nm to hundreds µm [7,17,24]. Different is the case of parent alloys in the shape of thin films, and micro- and nanowires. In these cases, fabrication usually involves deposition methods such as sputtering or physical vapor deposition (PVD), processes that lead to smaller grain sizes. An exception is represented by the commonly called *gold leaf*, an Au-Ag thin foil with a thickness down to 100 nm, obtained by hammering thicker rolled foils. In this case, the alloy is prepared by the common melting, casting, and annealing before the mechanical thinning. Thus, the leaf presents grain sizes comparable to those of macroscopic bulk samples.

During and after dealloying, the material preserves the crystal orientation and grain size of the parent alloy, leading to a NP metal with the same crystal orientation and grain size of the parent alloy, although the precursor alloys with a different type of unit cell or amorphous alloys can display a different behavior in this regard. The influence of the alloy type on the thermal stability of the NP Au is the main scope of the present study as far as an analysis of literature data is performed.

To further clarify the role of the microstructure on the thermal stability of the NP Au, we investigated the behavior of NP Au produced from Au-Ag precursor alloys prepared by mechanical alloying (MA).

MA allows fabricating metastable alloys with particularly fine microstructures that can be used to obtain NP metals with unusual chemical compositions and properties. MA is performed on elemental powders by ball milling. The produced alloy powders can then be pressed and sintered in different ways. The use of powder metallurgy has several advantages compared to the common metallurgical processes, since it minimizes waste production, facilitates the manufacture of products with unique or complex shapes, which would be impossible in the other cases, is easily scalable from 10^−4^ to 20 m^3^ for industrial production, does not require high-skilled operators, and is cost-effective for mass production. At the same time, MA generally leads to nanocrystalline materials [25]. Since dealloying does not modify the microstructure of the parent alloy [6], the resulting NP metal is nanocrystalline as well. This is how we produced a nanocrystalline NP Au from the mechanically alloyed precursor. We find that the NP Au produced in this way presents a lower thermal stability of the porous structure, which can be enhanced by performing thermal annealing before dealloying.

## 2. Materials and Method

### 2.1. NP Au Fabrication

#### 2.1.1. Au-Ag Alloy Fabrication

An amount of 2 g of Au-Ag powder mixture was prepared in an atomic ratio of 30:70. The powders were mechanically alloyed in a SPEX Mixer/Mill 8000 ball mill for 16 h using a hardened steel vial with two 8 g hardened steel balls. The powders were removed from the sides of the vial every 30 min in the first 2 h, and then every 5 h to keep the mixture homogeneous. Pellets of 13 mm of diameter and 1 mm of thickness were prepared by cold pressing under 10,000 kg for 5 min with a hydraulic press. Predealloying thermal treatments were conducted in an SN 388589 (Nabertherm GmbH, Bremen, Germany) oven in air at different temperatures, ranging from 750 °C to 950 °C, for times ranging from 5 h to 100 h.

#### 2.1.2. Dealloying of Au-Ag Alloy

NP Au was prepared by chemical dissolution of Ag in concentrated HNO_3_ for 24 h. During the treatment, the acidic solution was repeatedly stirred. After the treatment, the pellets were rinsed three times and stored in ultrapure water for 24 h. Then, the samples were dried in a desiccator.

#### 2.1.3. Post-Dealloying Annealing Treatments

The dealloyed NP Au samples were subjected to thermal treatments at 100 °C, 200 °C, 300 °C, 400 °C, and 600 °C for 1 h in an SN 388589 (Nabertherm GmbH, Bremen, Germany) oven in air. The samples were quickly inserted into the oven when it had reached a stable target temperature. 

### 2.2. Scanning Electron Microscopy Measurements

Scanning Electron Microscopy (SEM) was carried out with an S400 (Hitachi, Tokyo, Japan) scanning electron microscope equipped with an Everhart–Thornley secondary electrons detector and an UltraDry EDS detector (Thermo Fisher Scientific, Waltham, MA, USA). 

### 2.3. X-ray Diffraction Measurements

The X-ray Diffraction (XRD) measurements were carried out with a *Miniflex II* diffractometer (Rigaku, Tokyo, Japan) with a Bragg–Brentano geometry working in a θ−2θ configuration. Quantitative phase and microstructure analysis were performed through an extended Rietveld refinement method [26,27] using MAUD software(v. 2.933, Radiographema, Trento, Italy).

### 2.4. Image Analysis

The SEM images were collected and analyzed from our measurements and the literature [19,20,21,22,23,28,29,30,31,32,33,34,35,36,37,38,39,40,41] with Fiji [42]. Binary images were obtained by converting solid and voids into 1 and 0 values, and then different procedures were applied to obtain the microstructural information:

- Ligament diameters were measured manually through the *Measure* function;
- Pore diameters, pore densities, the perimeter per unit of surface, and the density ϕ of the material were measured through the function *Analyze Particles*. In this algorithm, pores are fitted as ellipses, and the mean diameter is estimated as the average between major and minor axis of the ellipse. In particular, the density ϕ of the material was estimated by the ratio between the number of the pixels with a value of “1” and the number of all pixels of the examined area.

**Figure 1:**
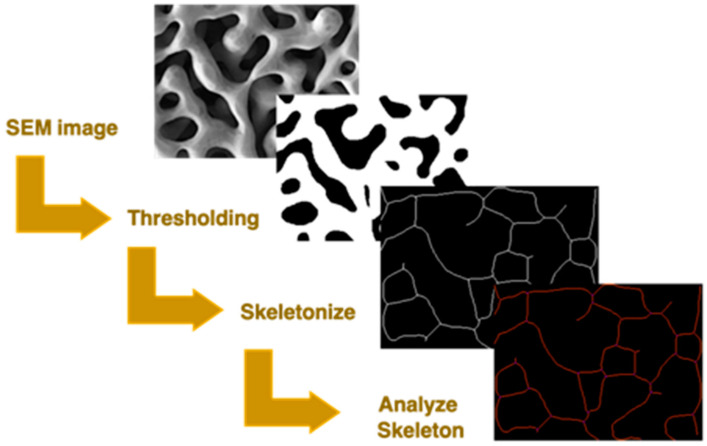
Scheme of image analysis by thresholding, skeletonization, and successive analysis through Fiji.

Ligament lengths, ligament, and node density were measured by converting the binary image in a skeleton of branches and nodes using the *skeletonize* function. The output was analyzed through the *analyze skeleton* function. The process is illustrated in Figure 1.

## 3. Results and Discussion

### 3.1. Literature Image Analysis

Several publications reported that coarsening can induce a densification of NP Au samples. Densification becomes predominant under certain annealing conditions and it can even determine the complete collapse of the NP structure [20,22,38]. The analysis of the literature data unveils a first, common feature of NP Au coarsening, namely that major structural changes take place at temperatures higher than 300 °C, regardless of the length of thermal treatment. Therefore, we selected the reports concerning NP Au samples exposed to temperatures equal to or higher than 300 °C. A total of 19 publications satisfy this requirement [19,20,21,22,23,28,29,30,31,32,33,34,35,36,37,38,39,40,41]. Within this group, we found 8 cases in which densification is prominent [20,22,23,32,37,38,40,41] (group *i*) and 11 in which no densification is observed or, if any, kept almost negligible [19,21,23,28,29,30,31,33,35,36,39] (group *ii*). Within group *i*, five cases, i.e., 63%, involve thin films, nanowires, or nanoparticles [20,22,38,40,41], while two cases concern bulk Au-Ag alloys [23,32], and one case considers a bulk AuAgCuNiZn alloy, with no information on its microstructure [37]. As far as group *ii* is concerned, 82% of the reports involve bulk Au-Ag alloys [19,29,30,31,33,34,35,36,39], where case concerned a 100 nm thick Au leaf with coarse grains [21] and one case focused on an Au leaf with an intermediate thickness of 1 µm [28]. However, the morphology of the ligaments and pores does not show any specific correlation with the dimensionality of the macroscopic sample, so that bicontinuous and tunable structures can be produced from 0D, 1D, 2D, and 3D materials [19,22,41,43,44]. These observations suggest that densification mostly depends on the solid dimensionality and not on the NP structure. The different behaviors can be caused by stresses that arise from geometrical constraints, which lead to shrinkage and the consequent filling of the pores. Moreover, the grain size of low-dimensional samples are commonly smaller than those of molten and casted alloys due to the different preparation methods. Moreover, the only two cases of thin films stable against densification were made from a coarse-grained precursor, but there was also a case in which, even if the structure did not collapse, the Au leaf densified [20]. Thus, the evidence is not sufficient to draw any conclusion about the factors determining densification.

### 3.2. Experimental Findings on Microstructure-Related Thermal Stability

**Figure 2:**
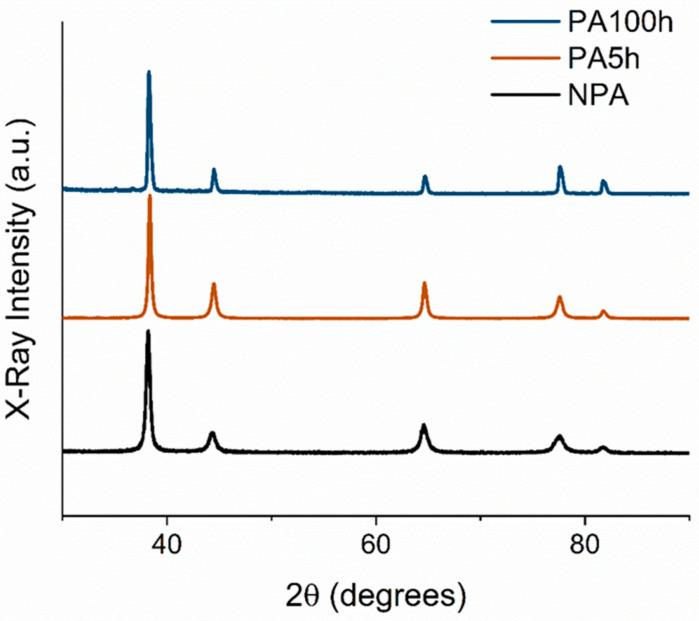
XRD pattern of NPA and PA samples.

With the purpose of better understanding the role of the microstructure, we studied how it affects the thermal stability of the NP Au by carrying out suitably designed experiments. NP Au samples were produced starting from nanocrystalline Au_30_Ag_70_ alloys fabricated by MA. The parent alloys were subjected to annealing prior to dealloying precisely to investigate the effects of annealing on the NP Au properties. The XRD measurements on the pristine (P) parent alloys and parent alloys that were annealed for 5 h (A5h) and for 100 h (A100h) are reported in Figure 2. It can be seen that the XRD spectra exhibit the characteristic peaks of Au and Ag phases. Rietveld refinement was performed on each pattern to evaluate the grain size of the alloys. The P alloy shows a crystallite size around 30 nm, while the A5h and A100h have grain sizes around 50 nm and 85 nm, respectively.

**Figure 3:**
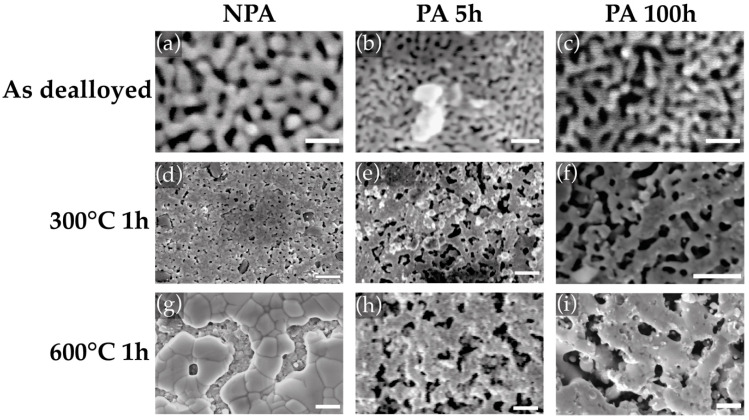
SEM images of NPA NP Au as prepared (**a**) after thermal treatment for 1 h at 300 °C (**d**) and 600 °C (**g**); SEM images PA5h NP Au as prepared (**b**) after thermal treatment for 1 h at 300 °C (**e**) and at 600 °C (**h**); SEM images PA100h NP Au as prepared (**c**) after thermal treatment for 1 h at 300 °C (**f**) and at 600 °C (**i**). Scale bars: (**a**–**c**): 100 nm; (**d**–**i**): 1 µm.

**Figure 4:**
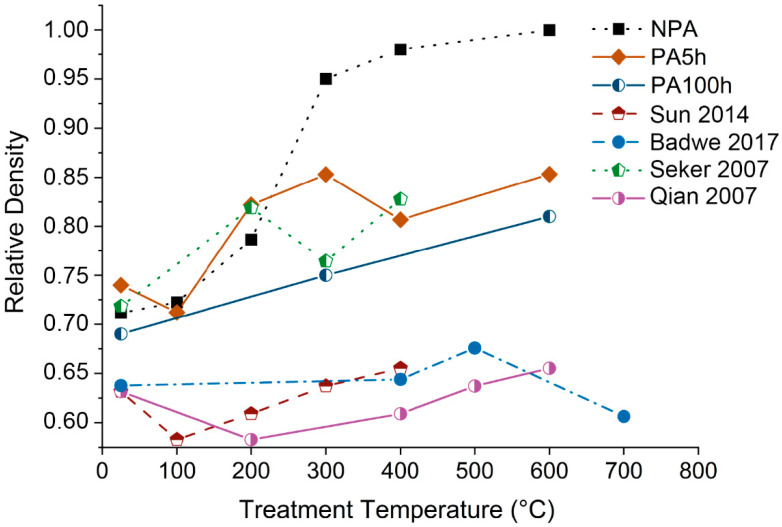
Density trends over annealing temperature of our samples and the literature ones. The samples in the selected reports were submitted to annealing for 10 min (Seker et al. [22] and Sun et al. [23]), 15 min (Badwe et al. [39]), and 120 min (Qian et al. [35]).

The samples were subjected to dealloying in HNO_3_ 70% for 24 h. The SEM images of the resulting materials are shown in Figure 3a–c. The samples present a bicontinuous NP structure with ligament diameters around 30 nm, ligament lengths around 50 nm, and pore diameters around 40 nm. Moreover, the annealing did not result in any significant variation of the NP structure, which maintains its bicontinuity up to 300 °C. The NP Au images after the thermal treatments for 1 h at 300 °C and 600 °C are shown in Figure 3d–i for both the P and A samples. At 300 °C, important differences can be found between the P and A NP structures. The P samples show significant densification from an initial ϕ value of 70% to almost 100% for the sample annealed at 600 °C. At 300 °C, the NP structure is already collapsing. The observations are confirmed by IA results, shown in Figure 4. Nevertheless, the A5h samples show enhanced thermal stability, undergoing limited densification. Moreover, a significant enhancement in thermal stability can be obtained by prolonging the annealing duration from 5 h to 100 h. In this case, the morphology of the sample treated at 300 °C for 1 h, shown in Figure 3f, looks closer to the usual bicontinous NP structure, while there is no significant difference between the two P samples after the thermal treatment at 600 °C. In addition, Figure 4 shows that the density is further reduced from 85% to 75% in the sample treated at 300 °C and from 85% to 81% in the sample treated at 600 °C.

The annealing effect on thermal stability is therefore fundamental for preserving the NP structure at high temperatures. The beneficial effect of annealing the parent alloys on the thermal stability of the NP Au structure can be ascribed to a decrease of the grain boundary concentration due to the larger grain size of the A5h NP Au of about 67% and of the A100h NP Au of about 183%. These are significant differences at this scale, where the grain boundary density rapidly changes with grain size [45]. Additionally, it is known that the grain boundary diffusion causes densification [46,47,48] while the surface diffusion does not [49]. Therefore, lowering the density of the grain boundaries probably leads to a less efficient densification in the P samples. 

**Figure 5:**
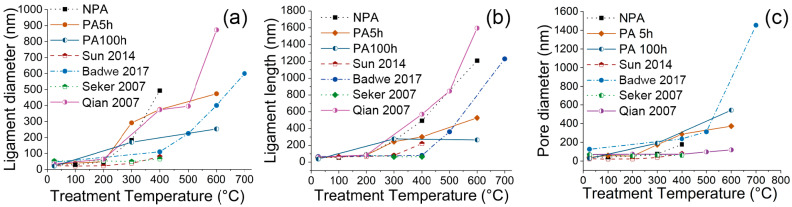
Values of ligament diameter (**a**), ligament length (**b**), and pore diameter (**c**) at different treatment temperatures from image analysis of our samples and the literature ones [22,23,35,39].

The evolution of the pristine NP Au samples has been compared with the data taken from the selected publications. Figure 4 shows, in particular, the evolution of the density at different temperature values, while Figure 5 shows how the ligament diameter and length, and pore diameter, change with temperature. For convenience, only some representative trends were plotted together with those observed in our samples. Specifically, we show those of the 3D samples investigated by Badwe et al. [39], Sun et al., and Qian et al. [35], and of the thin microbeams of NP Au fabricated by Seker et al. [22]. The P NP Au we prepared exhibits much lower stability compared to commonly produced NP Au examples. The P NP Au display ligament and pore sizes are comparable with those reported in the literature, while it still shows pronounced densification compared to the 3D samples of the NP Au produced from Au-Ag alloys by melting and casting. Thus, the result is more similar to the behavior observed by Seker et al. in their fabricated microbeams [22].

The larger density increase of the P NP Au compared with the majority of 3D NP Au samples can be ascribed to grain sizes that are still smaller than those of the common NP Au. These observations confirm that the microstructure deeply affects the NP Au structure at high temperatures, while it does not have significant effects on the morphology during dealloying. Furthermore, the work shows that the thermal stability of NP Au produced by MA depends on temperature treatments made before the dealloying. It is thus necessary to optimize this procedure to further reduce the densification in the material in potential applications at high temperatures. It is probably necessary to achieve grain sizes larger than the ligament sizes in order to make surface diffusion dominant during coarsening compared to grain boundaries diffusion.

## 4. Conclusions

On the basis of the analysis of data available in literature and addressing specific issues with suitably designed experiments, this work focuses on the coarsening process undergone by NP Au. Our results show that annealing in NP Au induces densification more easily in systems presenting short characteristic lengths. The NP Au obtained by the dealloying of mechanically prepared AuAg parent alloys exhibit lower thermal stability of its porous structure compared with that of several NP Au samples reported in the literature. However, NP Au samples prepared by annealing the precursor alloy before dealloying display a reduction of ligaments and pores coarsening and a decrease of densification. Nevertheless, also in this case, densification is more pronounced than in monolithic coarse-grained NPs Au, which are obtained by Au-Ag alloys prepared by melting and casting. The contrasting behaviors are probably due to the different grain sizes presented by the differently prepared samples. In particular, the smaller the grain size of the parent alloy, the lower the thermal stability.

## Data Availability

Not applicable.
